# Education and health equity: diverse learning needs in the modern era

**DOI:** 10.1016/j.lanwpc.2026.101846

**Published:** 2026-03-31

**Authors:** The Lancet Regional Health – Western Pacific

“Education is the kindling of a flame, not the filling of a vessel.”

Socrates

This notion, emphasising critical thinking, curiosity, and creativity is more important than ever in our era of globalisation and digitalisation. While digitalisation offers many benefits such as personalised learning and improved efficiency, it also raises important questions about the future of learning and employment, and ultimately, aspects of the human condition that are truly unique.

On March 2, 2026, the Australian Government announced a major educational reform redefining education equity by prioritising diverse learning needs and moving beyond mere education access. The Inspire program seeks to provide access to high potential and gifted education across all public schools in Australia, which has mostly been available in private or selective schools. High potential and gifted education addresses diverse learning needs by offering tailored support to children with strong abilities or potential in four domains (intellectual, creative, physical, and social–emotional), regardless of their background. Truly equitable education means quality education that nurtures individual growth, ensuring every child can reach their full potential and achieve optimal health and wellbeing. “It's about equity, not elitism.”

Education influences health literacy, employment opportunities, income levels, and access to health care and healthy foods, as well as safe living environments. The average years of schooling vary considerably within the Western Pacific region, including by sex and rural or remote residence. For example, children spend 9.0 years in schooling in Papua New Guinea, 12.5 years in China, and 13.2 years in Australia. Globally, adults with 12 years of schooling have an approximately 24.5% lower risk of death compared with adults with no schooling, including in high-income Asia–Pacific countries in which individuals with primary level education have more than double the risk of death compared with college graduates. In China, people with less than primary education have nearly twice the risk of premature death, especially from respiratory diseases. Women and people living in urban areas have the highest risks, with income mediating 23.0% of the effect in men and 11.1% in women. There may be a generational effect, by which educational inequalities in mortality have grown larger between those born in the 1940s and those born in the 1970s in China, particularly in rural areas. Almost half of the link between education level and mortality across all generations were mediated by socioeconomic, behavioural, and metabolic factors, but the influence of these factors decreased in younger generations, with physical activity playing a greater role.

These individual and generational differences highlight that access to education alone is not enough; quality education that focuses on diverse learning styles, abilities, cultures, and personal experiences are vital. Considering the broader health impacts of education and adopting a more balanced approach that supports both academic success and wellbeing are essential. For example, academic pressure, especially from private tutoring in some Asian countries (referred to as juku in Japan and hagwon in South Korea) has raised concerns about stress and burnout, as well as increased risk of myopia. Older Taiwanese adults with fewer years of education (≤12 years) and Japanese children whose parents had lower educational attainment may also be at higher risk of overweight and obesity. Childhood is a critical age window for health development; young people are developing habits and behaviours that can last their lifetime. The school environment is therefore ideal to shape positive health behaviours and reduce disparities at the outset. This can include greater access to vaccinations and mental health programmes. The Health Promoting Schools framework in the Western Pacific region advocates for an integrated approach to health and education. For example, curriculums including nutrition, physical activity, mental health awareness, and water, sanitation, and hygiene have been implemented in public primary schools in Manila, Philippines.

Education influences health beliefs, behaviour, and outcomes throughout one's life. We must think critically and creatively to transform the educational landscape. Prioritising diverse learning needs provides a valuable opportunity to tailor the future of education, ensuring that all children can access and meaningfully benefit from learning. In this way, children become active participants in their learning and health journeys empowered with the means to thrive in a rapidly changing world. In turn, we will truly bridge the gap between education and health equity to build resilient communities in the Western Pacific.

